# Urine cytological study in patients with clinicopathologically confirmed neuronal intranuclear inclusion disease

**DOI:** 10.3389/fnagi.2022.977604

**Published:** 2022-09-12

**Authors:** Yiyi Zhou, Pengcheng Huang, Zhaojun Huang, Yun Peng, Yilei Zheng, Yaqing Yu, Min Zhu, Jianwen Deng, Zhaoxia Wang, Daojun Hong

**Affiliations:** ^1^Department of Neurology, The First Affiliated Hospital of Nanchang University, Nanchang, China; ^2^Department of Neurology, Peking University First Hospital, Beijing, China; ^3^Department of Medical Genetics, The First Affiliated Hospital of Nanchang University, Nanchang, China

**Keywords:** neuronal intranuclear inclusion disease, skin biopsy, urine cytology, *NOTCH2NLC* gene, pathological diagnosis

## Abstract

**Objective:**

The diagnosis of neuronal intranuclear inclusion disease (NIID) is currently based on CGG repeat expansion in the 5′UTR of the *NOTCH2NLC* gene, or p62-positive intranuclear inclusions in skin biopsy. The purpose of this study is to explore the value of non-invasive pathological findings in urine sediment cells from NIID patients.

**Materials and methods:**

Ten patients with clinically suspected NIID were enrolled for skin biopsy and gene screening. Morning urine (500 ml) was collected from each patient, and cell sediment was obtained by centrifugation. Urine cytology, including Giemsa staining, p62 immunostaining, and electron microscopic examination, were conducted on cell sediment.

**Results:**

The main clinical symptoms of 10 patients included episodic disturbance of consciousness, cognitive impairment, tremor, limb weakness, and so on. Cerebral MRI showed that 9 patients had linear DWI high signal in the corticomedullary junction. Genetic testing found that the number of CGG repeat ranged from 96 to 158 in the *NOTCH2NLC* gene. Skin biopsy revealed that all patients showed p62-positive intranuclear inclusions in 18.5 ± 6.3% of the duct epithelial cells of sweat gland. In contrast, urine sediment smears revealed that only 3 patients had p62 positive intranuclear inclusions in 3.5 ± 1.2% of the sedimentary cells. Ultrastructural examinations showed that intranuclear inclusions were also identified in the cell sediment of the 3 patients.

**Conclusion:**

Urine cytology may be a new and non-invasive pathological diagnosis technique for some NIID patients, although the positive rate is not as high as that of skin biopsy, which is a sensitive and reliable pathological method for NIID.

## Introduction

Neuronal intranuclear inclusion disease (NIID) is a rare neurodegenerative disease, initially named after the pathological features characterized by eosinophilic intranuclear inclusions in neurons (Liufu et al., 2022). Further studies have found that eosinophilic intranuclear inclusions also exist in the cells of peripheral nervous system and the most other organs (Miki et al., 2022). Abnormal CGG repeat expansion in the 5′-untranslated region (5′UTR) of the *NOTCH2NLC* gene is the genetic cause of NIID (Deng et al., 2019; Ishiura et al., 2019; Sone et al., 2019; Tian et al., 2019). In addition, studies have shown that the repeat expansion is also associated with Parkinson’s disease (PD) (Shi et al., 2021), essential tremor (Sun et al., 2020), multiple system atrophy (Fang et al., 2020), motor neuron disease (Yuan et al., 2020), peripheral neuropathy (Wang et al., 2021), and oculopharyngodistal myopathy (OPDM) (Yu et al., 2021). Collectively, these phenotypes are referred to as *NOTCH2NLC*-related repeat expansion disorders (NREDs) (Lu and Hong, 2021). Among them, NIID is the most common subtype, and its clinical manifestations show great heterogeneities, such as sudden disturbance of consciousness, episodic psychiatric or cognitive impairments, seizures, limb weakness, tremor, miosis, and autonomic dysfunctions (Sone et al., 2016; Liang et al., 2020; Wang et al., 2020). Although the causative gene of the NIID has been identified, the relationship between the phenotype and genotype remains uncertain in some NIID patients. Therefore, pathological examination still plays a crucial role in the accurate and timely diagnosis of NIID (Chen et al., 2020b).

Skin biopsy has been developed as a sensitive and specific method for pathological diagnosis of NIID, which is characterized by p62-positive eosinophilic intranuclear inclusions in fibroblasts, adipocytes, and duct epithelial cells of sweat gland (Sone et al., 2011). As an invasive examination, open skin biopsy usually takes specimens deep into the dermis and subcutaneous adipose tissue, thus increasing the risk of potential infection and pain in patients. In addition, the genetic screening is expensive and unavailable to most patients. Therefore, it is necessary to find a non-invasive and economic examination for the diagnosis of NIID patients. In this study, 10 patients with NIID were diagnosed according to the clinical, radiological, pathological and genetic characteristics, and then the urine of 10 patients was collected in the acute phase of hospitalization to explore whether urine cytology was helpful to the diagnosis of NIID.

## Materials and methods

### Subjects

A total of 10 NIID patients who were referred to the Department of Neurology, the First Affiliated Hospital of Nanchang University were recruited from January 2021 to February 2022. The clinical features and radiological data of the patients were collected, and their family history and symptoms of family members were obtained from the subjects and their relatives. This study was approved by the Ethics Committee of The First Affiliated Hospital of Nanchang University. The tissue samples of the patients and controls were obtained under a written consent signed by each individual in compliance with the bioethics laws of China as well as the Declaration of Helsinki.

### Genetic screening

Peripheral blood was taken from each patient in 3 ml for DNA extraction. Repeat-primed polymerase chain reaction (RP-PCR) was initially used to identify the repeat expansion in the *NOTCH2NLC* gene. RP-PCR was performed as described in our previous study (Deng et al., 2019). The PCR primer mix contained three primers: NOTCH2NLC-F:5′-FAM-GGCATTTGCGCCTGTGCTTCGGACCGT-3′, M13-(GGC)4 (GGA)2-R:5′-CAGGAAACAGCTATGACCTCCTCCGCCGC CGCCGCC-3′, and M13-linker-R: 5′-CAGGAAACAGCTA TGACC-3′. A saw-tooth tail pattern in the electropherogram was considered to be the disease-associated repeat expansion. Fluorescence amplicon length polymerase chain reaction (AL-PCR) was used to detect the length of GGC repeat expansion. The composition of PCR mix was identical to that of RP-PCR except for the use of 50 ng genomic DNA as a template and a different primer pair: NOTCH2NLC-AL-F: 5′-VIC-CATTTGCGCCTGTGCTTCGGAC-3′; NOTCH2NLC-AL-R: 5′-AGAGCGGCGCAGGGCGGGCATCTT-3′. The PCR conditions were the same as for RP-PCR. Electrophoresis was performed on a 3500xl Genetic analyzer (Thermo Fisher Scientific, Waltham, MA, United States) and the data were analyzed using GeneMapper software (Thermo Fisher Scientific). The length of the highest signal peak of expanded allele was used to calculate the repeat number.

### Pathological examination of skin biopsy

Skin biopsy in the distal part of the leg (10 cm above the external malleous) was performed in the 10 patients. A part of the specimen was fixed by 4% formalin solution, embedded in paraffin, cut into 4-mm thick sections, and stained with hematoxylin and eosin (H&E). The immunohistochemical and immunofluorescent staining were performed with anti-p62 antibody (sc-28359, Santa Cruz Biotechnology, CA, United States). The rate of p62-positive intranuclear inclusions was calculated by the number of positive cells to the total number of the duct epithelial cells of sweat gland. For electron microscopy, a portion of the specimens were initially fixed in 2.5% glutaraldehyde, subsequently fixed in 1% osmium tetroxide, and embed in Epon 812. Ultrathin sections were examined through electron microscope (JEOL-1230, Japan).

### Pathological examination of urine sediment

Five hundred milliliters of morning urine were collected in the 10 NIID patients and 6 healthy controls, subsequently centrifuged at 1500 rpm/min for 5 min, discarded the supernatant, and collected the urine sediment into a cell cryopreservation tube. Suspending a small amount of sediment with phosphate buffered saline (PBS) for suspension, and then 100 μl of the suspension was drawn and dropped on the glass slide for natural drying, and were used for Wright’s Giemsa staining and anti-p62 immunostaining, respectively. The rate of p62-positive intranuclear inclusions was calculated by the number of positive nuclei to the total number of the nuclei on six random 100× microscopic fields. The remaining urine sediment were centrifuged at 5000 rpm/min for 5 min, discarded the supernatant. 1 ml 2.5% glutaraldehyde solution was added to the urine sediment for fixation, subsequently embedded in epoxy resin, stained with uranyl acetate and lead citrate. Ultrastructural pathological changes were observed under transmission electron microscope (JEOL-1230, Japan).

## Results

### Clinical features

Half of the patients (5/10) had family history (only the probands included), and 5 (5/10) patients were sporadic cases, including 4 male and 6 female patients. The age of onset was 54–73 (65.90 ± 4.95) years old, and the disease duration was 0.5–20 (6.95 ± 6.90) years. The presenting symptoms included episodic disturbance of consciousness (4/10), episodic headache (2/10), tremor (2/10), cognitive impairment (1/10), and episodic psychiatric disorder (1/10). The episodic disturbance of consciousness or mental disorder usually lasted from a few hours to several weeks, and most of them could return to their pre-onset state. Episodic headaches were similar to migraine attacks, lasting from a few hours to a few days, and usually relieved within a day. The main neurological clinical symptoms at visit included cognitive impairments (7/10), tremor (4/10), limb weakness (4/10), bradykinesia (2/10), psychiatric symptoms (2/10), sensory dysfunction (2/10), visual dysfunction (2/10), and seizures (1/10). Other multi-system symptoms included urinary dysfunction (4/10), dry cough (3/10), episodic fever (3/10), constipation (3/10), episodic abdominal pain (2/10), episodic nausea/vomiting (2/10), and diabetes (2/10). Physical examination revealed paresthesias in 4 patients (4/10), hyporeflexia of lower limb in 4 patients (4/10), decreased muscle tone in 4 patients (4/10), increased muscle tone in 3 patients (3/10), pyramidal signs in 3 patients (3/10), cerebellar ataxia in 2 patients (2/10), and miosis in one patient (1/10) (Table 1).

**TABLE 1:** Clinical data of 10 patients with neuronal intranuclear inclusion disease (NIID).

Variables	P1	P2	P3	P4	P5	P6	P7	P8	P9	P10
Age (years)	70	54	69	73	66	67	64	63	64	69
Sex	M	F	F	M	F	F	M	F	M	F
Disease duration (years)	3	3	2	5	17	20	2	15	2	0.5
Family history	+	+	+	+	+	−	−	−	−	−
Cognitive impairment	+	+	+	+	−	−	+	+	+	−
Tremor	−	+	−	−	+	+	+	−	−	−
Limb weakness	−	−	−	+	+	+	−	−	−	+
Bradykinesia	−	−	−	−	−	−	−	+	+	−
Psychiatric symptoms	−	+	−	+	−	−	−	−	−	−
Sensory dysfunction	−	−	−	+	−	−	−	+	−	−
Visual dysfunction	−	+	−	−	−	−	−	−	−	+
Seizures	−	−	−	−	−	+	−	−	−	−
Urinary dysfunction	+	−	−	+	−	+	+	−	−	−
Dry cough	−	+	−	−	+	−	−	−	+	−
Episodic fever	+	−	−	+	−	−	−	+	−	−
Constipation	+	−	−	+	−	−	+	−	−	−
Episodic abdominal pain	−	−	−	+	−	−	−	+	−	−
Episodic nausea and vomiting	−	+	−	−	+	−	−	−	−	−
Diabetes	−	+	−	−	−	−	−	−	−	+
Paresthesia	−	−	−	+	−	+	−	+	−	+
Reduced reflex	−	−	−	+	−	+	−	+	−	+
Increased muscle tension	−	+	−	−	+	−	−	−	+	−
Pathological signs	−	+	−	−	+	−	−	−	+	−
Ataxia	−	−	−	−	−	−	−	+	+	−
Miosis	−	−	−	−	−	+	−	−	−	−

**FIGURE 1:**
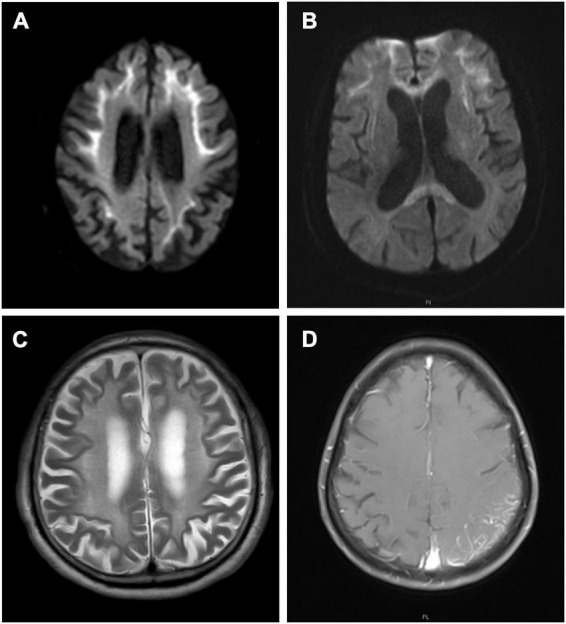
Cerebral magnetic resonance imaging (MRI) features of neuronal intranuclear inclusion disease (NIID) patients. Diffusion weighted imaging (DWI) showed curve-like hyperintensity along the corticomedullary junction **(A)**; DWI also showed lesions in the corpus callosum **(B)**; T2-weighted showed symmetrical white matter lesions **(C)**; Contrast T1-weighted showed the left temporal occipital cortical edema with enhancement **(D)**.

Nerve conduction studies revealed that all patients had different degrees of peripheral neuropathy, including demyelinating sensorimotor neuropathy in 6 patients (6/10), mixed sensorimotor neuropathy in 3 patients (3/10), and demyelinating sensory neuropathy in one patients (1/10) (Table 2). Cerebral magnetic resonance imaging (MRI) showed 9 patients (9/10) with abnormal curve-like hyperintensity along the corticomedullary junction on diffusion weighted imaging (DWI) (Figure 1A), and 2 patients (2/10) had involvements in the corpus callosum (Figure 1B). White matter hyperintensities were symmetrically observed in the corona radiata, the center of the semiovale, and the lateral ventricle in 9 patients (9/10) (Figure 1C). One patient (1/10) presented with edema of the left temporo-occipital cortex, which was significantly enhanced on contrast scan (Figure 1D).

**TABLE 2:** Electrophysiological changes in 10 patients with neuronal intranuclear inclusion disease (NIID).

*P*	Age (y)*	Motor NCV												Sensory NCV					

		Median nerve			Ulnar nerve			Peroneal nerve			Tibial nerve			Median nerve		Sup peron. nerve		Sural nerve	
								
		DML (≤4.0 ms)	CV (≥50 m/s)	CMAP (≥4.0 mV)	DML (≤3.0 ms)	CV (≥50 m/s)	CMAP (≥3.5 mV)	DML (≤5.3 ms)	CV (≥40 m/s)	CMAP (≥2.0 mV)	DML (≤5.0 ms)	CV (≥40 m/s)	CMAP (≥3.5 mV)	CV (≥50 m/s)	SNAP (≥5 μV)	CV (≥40 m/s)	SNAP (≥1 μV)	CV (≥40 m/s)	SNAP (≥1 μV)
1	70	3.7	45	7.4	3.3	49	8.2	4.9	55	3.3	4.5	36	6.5	46	3.5	NA	NA	38	4.8
2	54	4.3	47	4.8	3.5	48	5.5	3.8	38	3.0	NA	NA	NA	40	5.7	NA	NA	NR	NR
3	69	3.6	48	5.3	2.1	55	8.8	4.7	36	2.7	4.5	33	8.8	46	2.3	44	2.6	NA	NA
4	73	4.2	50	6.4	2.9	52	7.2	5.9	37	0.1	4.0	36	7.3	45	16.7	NA	NA	48	6.7
5	66	4.1	33	3.3	3.0	37	2.7	6.5	26	0.8	6.7	29	1.5	38	3.1	NR	NR	30	0.9
6	67	4.3	52	5.5	2.8	51	7.1	3.8	40	5.5	4.5	40	5.7	54	11.9	42	2.8	35	4.5
7	64	3.6	38	3.5	2.7	40	3.4	4.1	38	1.5	3.7	34	4.2	39	9.3	27	3.2	32	5.9
8	63	3.3	45	6.6	3.6	47	4.2	3.8	33	3.6	NA	NA	NA	48	4.8	NR	NR	NR	NR
9	64	4.7	37	3.8	2.5	50	4.5	3.9	35	1.4	4.4	36	3.4	35	4.2	42	4.6	NA	NA
10	69	3.9	48	9.7	2.6	53	5.7	6.0	37	2.6	6.5	39	3.8	46	6.4	31	4.2	NA	NA

*Age at examination; reference value at age of 65 years in our lab. CMAP, compound motor action potential; CV, conduction velocity; DML, distal motor latency; SNAP, sensory nerve action potential; NA, not available; NR, no response; NCV, nerve conduction velocity.

### Genetic mutation

**FIGURE 2:**
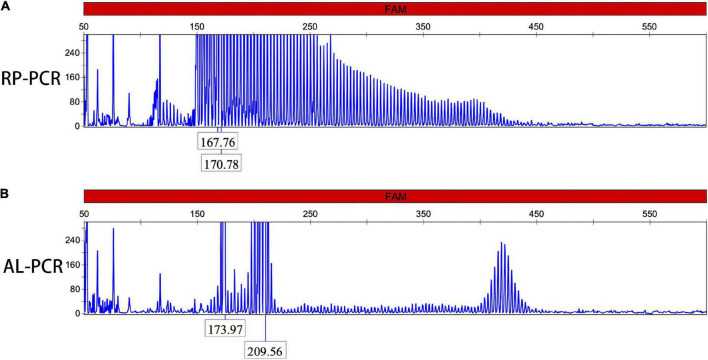
Dynamic variant of the *NOTCH2NLC* gene. Repeat-primed polymerase chain reaction (RP-PCR) chromatogram of patients 1 showed a long saw-tooth curve, indicating the presence of CGG repeat expansion **(A)**; Amplicon length polymerase chain reaction (AL-PCR) showed that the number of CGG repeat was 102 **(B)**.

Repeat-primed PCR amplification of the 5′UTR of the *NOTCH2NLC* gene revealed that the chromatograms of all patients showed long saw-tooth curves, indicating the presence of CGG repeat expansion variant (Figure 2A). In addition, the AL-PCR amplification showed that the number of CGG repeat in these patients ranged from 96 to 158, with an average number of 119 ± 23 (Figure 2B).

### Skin pathological features

**FIGURE 3:**
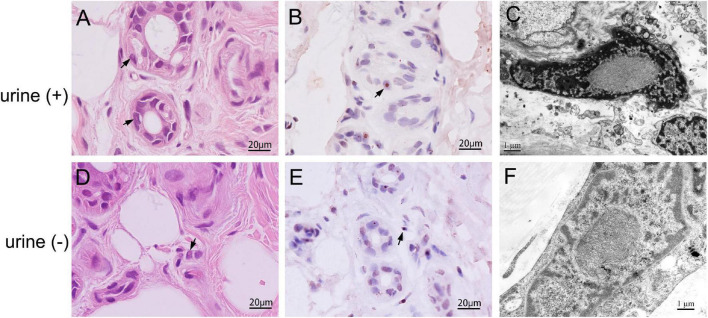
Skin pathology of neuronal intranuclear inclusion disease (NIID) patients with positive and negative urine sediment. H&E staining showed eosinophilic intranuclear inclusions in the nuclei of epithelial cells of the skin sweat gland ducts (Panels **A,D**, arrow); anti-p62 immunohistochemical staining showed positive intranuclear inclusions (Panels **B,E**, arrow); The intranuclear inclusions were seen as filamentous materials under electron microscope **(C,F)**.

Skin biopsies were performed in all 10 patients. The skin had no structural abnormalities and inflammatory cell infiltrations in the subcutaneous fatty tissue. Eosinophilic intranuclear inclusions were observed in the nuclei of fibroblasts, adipocytes, and duct epithelial cells of sweat gland (Figures 3A,D), and some of the inclusions presented with halo, especially in the nuclei of sweat gland duct epithelial cells. Occasionally, multiple inclusions were observed in a single nucleus. P62 antibody staining showed that positive intranuclear inclusions were observed in the nuclei of sweat gland duct epithelial cells, fibroblasts, and adipocytes in all patients (Figures 3B,E). The mean rate of p62-positive intranuclear inclusions accounted for 18.5 ± 6.3% of the duct epithelial cells of sweat gland in all patients. Electron microscopy revealed that intranuclear inclusions included filamentous materials with no membrane components around or within them (Figures 3C,F).

### Pathological changes of urinary sediment

**FIGURE 4:**
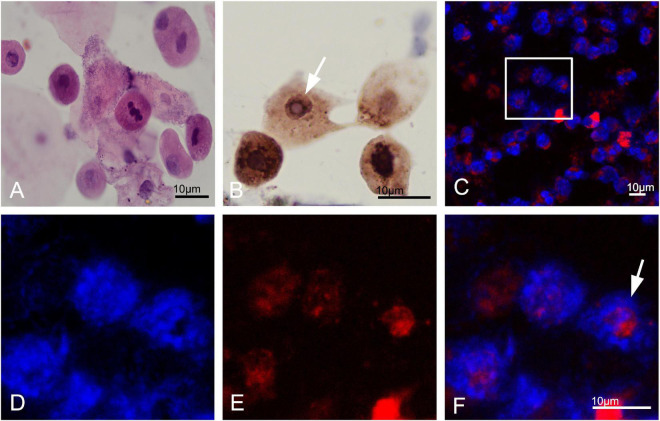
Urine cytology in neuronal intranuclear inclusion disease (NIID) patients. Leukocytes, epithelial cells, and epithelial cells were observed in the urinary sediment on Giemsa staining **(A)**. Immunohistochemistry showed p62-positive materials in the cytoplasm of sediment cells in patient 1 (Panel **B**, arrow). Immunofluorescence revealed that p62-positive intranuclear inclusions were observed in patient 1 (Panels **C–F** were magnification, arrow).

**FIGURE 5:**
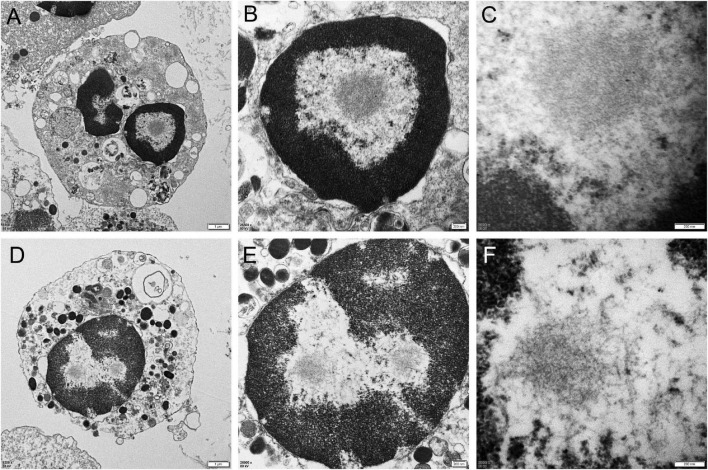
Electron microscopy of urine sediment cells. In the patient 1, electron microscopy showed the appearance of filamentous inclusions in a neutrophil (Panels **A–C** were magnification). In the patient 6, electron microscopy showed the appearance of filamentous inclusions in a monocyte (Panels **D–F** were magnification).

Different degrees of leukocytes, epithelial cells, erythrocytes, bacteria, and casts were observed in the urine sediment smear of all patients, while no intranuclear inclusions could be observed in the nuclei of the nucleated cells on Giemsa staining (Figure 4A). Five patients (5/10) received indwelling catheterization before or during hospitalization, and three patients (3/5) showed urinary tract infection in routine urine examination. Therefore, more urine sediments were collected from these 3 patients, and more nucleated cells were observed under the light microscope. Urine sediment smears of all patients were stained for p62 antibody through both immunohistochemistry and immunofluorescence. P62-positive materials could be observed in the cytoplasm of sediment cells in all patients and controls (Figure 4B), but p62-positive intranuclear inclusions were only observed in 3 patients with urinary tract infection (Figures 4C–F). The mean rate of cells with p62-positive inclusions accounted for only 3.5 ± 1.2% of the sedimentary cells in the 3 patients (3.2, 2.5, and 4.9%, respectively). The positive rate of skin biopsy was significantly higher than that of urine sediment (*p* < 0.01). Electron microscopy revealed filamentous inclusions in the nuclei of urinary neutrophils (Figures 5A–C) or monocyte (Figures 5D–F) in the patients.

## Discussion

In the period when autopsy pathology was the main diagnostic method, our understanding for the NIID was mainly limited to the nervous system (Cao et al., 2021; Fan et al., 2022; Huang et al., 2022). However, with the discovery of the NIID causative gene, the understanding about the disease was already beyond the scope of the previous cognition (Deng et al., 2019; Ishiura et al., 2019; Sone et al., 2019; Tian et al., 2019). In this study, the NIID patients showed great clinical heterogeneity, while deep analysis of the phenotypic features of the patients revealed some clues to the diagnosis. First, episodic symptoms were very common in our patients. Seven patients presented with episodic symptoms, including sudden disturbance of consciousness, sudden psychiatric disorder, and sudden headache, and some patients also experienced multiple episodes of fever, abdominal pain, and nausea and vomiting in the course of the disease. The duration of these episodic symptoms could vary greatly, but the symptoms were reversible after symptomatic treatment. Second, all patients in this cohort showed varying degrees of peripheral neuropathy. Some patients had no clinical symptoms of peripheral neuropathy, but electrophysiological studies exhibited that there were length-dependent sensorimotor demyelinating neuropathy, mainly characterized by mild NCV reduction and latency delay (Liao et al., 2022). Demyelinating impairment in these patients was more extensive and severe than axonal impairment, which might partly explain the absence of obvious neuropathy symptoms in some NIID cases. Third, in addition to the prominent neurological symptoms, these patients also showed multi-system symptoms, such as dry cough, paroxysmal abdominal pain, and paroxysmal nausea and vomiting (Chen et al., 2020a). Finally, the symptoms in some NIID patients were non-specific. Whether it was paroxysmal symptoms, autonomic nervous disorders or other visceral symptoms, they were easily neglected in the context of chronic degenerative diseases. In fact, the timely diagnosis in most our patients benefited from the characteristic features of cerebral MRI rather than the clinical phenotype.

In this study, nine of ten patients showed curve-like DWI high-intensity along the corticomedullary junction mainly in the fronto-parietal lobe, which was common in adult NIID patients, and also a specific radiological biomarker for the clinical diagnosis of NIID patients (Sone et al., 2014). The pathological basis of these imaging change may be associated with the progressive spongiform degeneration of subcortical U fibers (Yokoi et al., 2016). Our patients also presented with symmetrical distribution of white matter lesions, which were thought to be closely associated with oligodendrocyte degeneration. Abnormal CGG repeat expansion in the *NOTCH2NLC* gene was a major cause of non-vascular white matter lesions, so the characteristics of white matter lesions should be emphasized when interpreting the images of NIID patients (Liu et al., 2022). Meanwhile, two patients had persistent DWI hyperintensities in the corpus callosum. Previous study had reported this imaging feature, which might be related to degeneration of the large projective fibers of the corpus callosum (Wang et al., 2020). Notably, one patient did not have curve-like DWI high intensity and no white matter lesions, but temporo-occipital cortex edema and enhancement were the main image changes, similar to mitochondrial encephalomyopathy. Previous studies had reported a few cases of this distinct subtype of NIID (Liang et al., 2020).

Since the discovery of CGG repeat expansion in the 5′UTR of *NOTCH2NLC*, there have been more and more reports of expansion mutation, and the reported cases were mainly concentrated in Asia rather than Europe (Chen et al., 2020b). At present, it was believed that the number of CGG repeat in *NOTCH2NLC* was less than 40 in the normal controls. The number of CGG repeat between 41 and 60 was intermediate and might be associated with a few Parkinson’s disease or essential tremor (Fan et al., 2022). The number of CGG repeat more than 60 was pathogenic, and the typical phenotype of NIID usually had about 120 repeats. The number of CGG repeat in the 10 patients was more than 60, and the average number was approximately to 120. Therefore it was necessary to measure the CGG repeat number to determine its pathogenicity in the genetic diagnosis of NIID patients. The repeat expansion in *NOTCH2NLC* also showed some rare clinical phenotypes, such as neurodegenerative dementia (Jiao et al., 2020), non-vascular leukoencephalopathy (Liu et al., 2022), motor neuron disease (Yuan et al., 2020), sensorimotor with autonomic neuropathy (Wang et al., 2021), distal motor neuropathy (Wu et al., 2022), as well as oculopharyngeal distal myopathy (OPDM) (Yu et al., 2021). Because of the small number of these cases, the relationship between the clinical phenotype and the number of CGG repeat had not been established, but distal motor neuropathy and OPDM were generally considered to have more repeats. In addition, some studies had shown that carriers with more than 300 repeats exhibited very mild symptoms or no symptoms (Deng et al., 2022).

Neuronal intranuclear inclusion disease was named after its pathological characteristics. Therefore, the pathological examination of the intranuclear inclusions was still considered as one of the indispensable procedures for diagnosing the disease, although the disease-causative gene had been cloned. Sone et al. (2011) found that there were eosinophilic inclusions in the nuclei of sweat gland duct epithelial cells, adipocytes, and fibroblasts in the skin biopsies of NIID patients, and their composition and structural characteristics were almost the same as those in the CNS (Sone et al., 2011). Subsequent studies also confirmed the high consistency between the intranuclear inclusions of skin cells and the abnormal CGG repeat expansion in *NOTCH2NLC* (Deng et al., 2019). Our study also confirmed this association between genetic mutation and skin pathology. Collectively, skin biopsy had become the most important pathological diagnosis method for NIID.

Due to the great heterogeneity of NIID, especially when patients lacked typical features, such as DWI high-intensity along the corticomedullary junction, we needed more evidence to support the diagnosis of NIID or distinguish the diseases from other neurodegenerative diseases. However, open skin biopsy should not be routinely performed for every patient clinically suspected as NIID. In addition, conventional genetic screening was expensive and time-consuming. Considering that there were eosinophilic inclusions in the nuclei of renal tubular epithelial cells, we intended to search the intranuclear inclusions in the exfoliative cells in urine sediment cells (Sone et al., 2016). Among the 10 NIID patients confirmed by genetic screening and skin biopsy pathology, we found typical intranuclear inclusions in urine sediment cells of 3 patients by immunostaining, but the cell type could not be determined based the cell morphology alone. We further confirmed the filamentous inclusions in the nuclei of neutrophils and monocytes from the urine sediment by electron microscopy. Although the sensitivity of urine sediment was relatively lower than that of skin biopsy, it showed a certain value of application as a non-invasive examination.

In this study, there were some limitations in the pathological examination of urinary sediment cells. (1) The quantity and quality of cell sediment of 500 ml urine were quite different in these NIID patients, which directly affected the quality of subsequent cell smears and electron microscope examination. It might improve the positive rate of examination to collect more urine for cell sediment. (2) Because of the small amount of urine sediment, we could not conduct quantitative analysis before preparing the cell smear, resulting in a great difference in the cell density of the smear. The number of smear cells in some patients was too sparse to be observed, but the number of smear cells in other patients was too crowded to interfere with the observations. This might partly explain the low positive rate of urine sediment cytology for the pathological diagnosis of NIID. (3) Since the protocol characteristics of the study design and the relatively insufficient number of NIID cases, the area under curve (AUC) of receiver operating characteristic curve (ROC) could not be calculated in this study. In order to evaluate the diagnostic significance of urine cytology, a large-sample controlled trial will be needed in the future. (4) We found that p62-positive intranuclear inclusions were only observed in the three patients with urinary tract infection or indwelling catheterization, indicating that this method had some limitations, and urinary tract infection could improve the quantity and quality of urine sediment cells. Conversely, the limitation might be improved in patients with urinary tract infection, if urine cytology was performed before antibiotic treatment. (5) Most nucleated cells on the urine smear showed p62-positive materials in the cytoplasm. The epithelial cells and inflammatory cells in urine might gradually degenerate and lead to an increase in p62 levels in the cytoplasm (Ruppert et al., 2015), although the exact mechanism of p62 cytoplasmic positivity needed to be further explored. The p62-positive cytoplasmic materials greatly interfered with the rapid determination of intranuclear inclusions. If some specific antibodies can easily identify cell types and sources of urine sediment, the positive rate of urine cytology may be further improved.

In summary, our study showed that NIID had great clinical heterogeneity, of which episodic symptoms were various and non-specific. Skin biopsy had almost identical diagnostic value to the CGG repeat expansion in the *NOTCH2NLC* gene. Cytological pathology, a non-invasive and convenient pathological examination, showed p62-positive intranuclear inclusions in urine sediment cells of some patients with urinary tract infection, although the positive rate of urine sediment was not as high as that of skin biopsy. The method of urine cytology needed to be further optimized to improve the positive rate, and the number of patients needed to be further expanded.

## Data availability statement

The datasets presented in this study can be found in online repositories. The names of the repository/repositories and accession number(s) can be found in the article/supplementary material.

## Ethics statement

This research was approved by Ethics Committee of the First Affiliated Hospital of Nanchang University. The patients/participants provided their written informed consent to participate in this study. Written informed consent was obtained from the individual(s) for the publication of any potentially identifiable images or data included in this article.

## Author contributions

YYZ and PH: manuscript writing and data management. ZH, YP, YLZ, and MZ: data collection, data management, methodology, and biopsy. YY: specimen processing. ZW and JD: genetic testing, resources, supervision, and funding. DH: supervision, conceptualization, research, writing, and funding. All authors contributed to the article and approved the submitted version.
